# The Potential of Adjusting Water Bolus Liquid Properties for Economic and Precise MR Thermometry Guided Radiofrequency Hyperthermia

**DOI:** 10.3390/s20102946

**Published:** 2020-05-22

**Authors:** Kemal Sumser, Gennaro G. Bellizzi, Gerard C. van Rhoon, Margarethus M. Paulides

**Affiliations:** 1 Department of Radiation Oncology, Erasmus MC—Cancer Institute, University Medical Center Rotterdam, 3015 GD Rotterdam, The Netherlands; g.bellizzi@erasmusmc.nl (G.G.B.); g.c.vanrhoon@erasmusmc.nl (G.C.v.R.); m.m.paulides@tue.nl (M.M.P.); 2 EM4C&C Laboratory, Center for Care & Cure Technology Eindhoven (C3Te), Department of Electrical Engineering, Eindhoven University of Technology, 5612 AZ Eindhoven, The Netherlands

**Keywords:** hyperthermia, MR thermometry, MRI guided interventions, dielectric properties, MRI properties, water bolus

## Abstract

The potential of MR thermometry (MRT) fostered the development of MRI compatible radiofrequency (RF) hyperthermia devices. Such device integration creates major technological challenges and a crucial point for image quality is the water bolus (WB). The WB is located between the patient body and external sources to both couple electromagnetic energy and to cool the patient skin. However, the WB causes MRT errors and unnecessarily large field of view. In this work, we studied making the WB MRI transparent by an optimal concentration of compounds capable of modifying T${}_{2}$* relaxation without an impact on the efficiency of RF heating. Three different T${}_{2}$* reducing compounds were investigated, namely CuSO${}_{4}$, MnCl${}_{2}$, and Fe${}_{3}$O${}_{4}$. First, electromagnetic properties and T${}_{2}$* relaxation rates at 1.5 T were measured. Next, through multi-physics simulations, the predicted effect on the RF-power deposition pattern was evaluated and MRT precision was experimentally assessed. Our results identified 5 mM Fe${}_{3}$O${}_{4}$ solution as optimal since it does not alter the RF-power level needed and improved MRT precision from 0.39 ${}^{{}^{\circ}}$C to 0.09 ${}^{{}^{\circ}}$C. MnCl${}_{2}$ showed a similar MRT improvement, but caused unacceptable RF-power losses. We conclude that adding Fe${}_{3}$O${}_{4}$ has significant potential to improve RF hyperthermia treatment monitoring under MR guidance.

## 1. Introduction

Clinical trials have shown that the clinical outcome and local control of chemotherapy and radiotherapy treatments can be enhanced with the addition of hyperthermia treatments [1,2,3,4,5]. Clinically demonstrated thermal dose effect relationship indicate that the efficacy of the hyperthermia treatment improves when achieving higher temperatures in the 40–44 ${}^{{}^{\circ}}$C range in the entire target, while preventing unacceptable temperature increases in healthy tissues, i.e., “hotspots”. Advances in hyperthermia technologies improved the delivery of the treatment [6,7,8]. Electromagnetic radiofrequency systems consists of multiple antennas organized in an annular array configuration and using constructive interference provides focused energy deposition at depth, whereby the location of the focus can be dynamical adapted using phase and amplitude control per antenna [9,10]. For deep pelvic heating, the frequency range is 70–120 MHz, for deep heating of head and neck tumors the frequency range is 400–600 MHz. A multi-functional water bolus is used to reduce the antenna size, cool the skin and obtain a preferential energy transfer to the tissue. These advanced heating systems are clinical applied in combination with hyperthermia treatment planning guided focus steering. However, the inhomogeneous, variable and subject dependent thermoregulation mechanisms make that real-time temperature monitoring is required for optimal guidance of the heating adaptations. Magnetic resonance thermometry (MRT) provides a non-invasive way to map relative temperature change pattern in the regions of interest [11,12]. The need for non-invasive temperature monitoring and the potential of MRT fostered the development of novel magnetic resonance imaging (MRI) compatible hyperthermia applicators [13]. Such integration of a hyperthermia device into an MR scanner is not a trivial task and is accompanied by additional requirements and needs for these hybrid devices [14,15,16]. One of the issues that arise is caused by the hyperthermia device’s water bolus (WB). While the WB is a trivial component in non-MRI compatible hyperthermia applicators, it causes an unnecessary increase in the imaging field of view, creates flow artifacts and skews pre-scan calibrations, resulting in temperature errors and a longer scan time [9]. Solving these WB induced issues may form a large step towards reaching the full potential of MRI guided hyperthermia treatments, but our knowledge on the optimum WB filling is incomplete.

The WB is a fluid filled flexible “bag” that can conform to the skin and covers the space between antennas and patient. De-ionized, i.e., demineralized, water is usually used as filling material due to its large availability, high biocompatibility, and low losses. The WB has a pivotal role in a radiofrequency (RF) hyperthermia applicator. The water matches the dielectric properties of the patient better and thus enables efficient transfer of the radiofrequency waves from the device’s antennas into the patient. The water is circulated to cool so called “hotspots”, i.e., locations with high power absorption that often occur near the patient’s skin. Water as a fluid also easily follows the body contour which is essential for impedance matching and surface cooling. It can also be used to cool the antennas. The water is also applied to reduce the effective dielectric value of the medium surrounding the antennas, which decreases the resonant antenna size considerably and enables including more antennas in the applicator [17]. Unfortunately, the water also comes at a cost when used for MR guided hyperthermia. Hydrogen in water is the main source of MRI signal in the patient and the imaging field of view needs to cover also the WB to prevent aliasing in the MR image. Also, pre-MRI-scan calibrations and MRI RF (B${}_{1}$) field shaping are distorted since also the signal in the large WB is optimized by these automatic routines. Moreover, water circulation results in flow artifacts in the differential MRT images. It would be ideal if these problems could be avoided by a WB that is (nearly) invisible to MRI while preserving its benefits for hyperthermia, i.e., keeping the desired matching and cooling characteristics.

WB MRI invisibility can be reached by several methods. A simple solution can be achieved by using heavy water (deuterium oxide, D${}_{2}$O = ${}^{2}$H${}_{2}$O) instead of demineralized water (H${}_{2}$O). Heavy water is invisible in ${}^{1}$H MRI while having the same electromagnetic properties [18]. However, the required volume to fill a typical WB is large and heavy water is very expensive. Another method is by altering the MRI method by inversion recovery (IR) techniques, which are widely used in MRI applications to suppress water or fat signal. Using an IR sequence to suppress the signal from the WB, however, this will also suppress the water signal in the body that is used to measure the proton resonance shift in MRT. Another way to selectively suppress the WB signal is by selective excitation of the region of interest using two dimensional RF pulses [19]. Grissom et al. recently showed that two dimensional RF pulses can be used to reduce temperature errors related to the water bath and water bath motion for transcranial MR-guided focused ultrasound (MRgFUS) ablation. They reported on average 43% improvement in temperature precision [20]. While this technique shows promise, it is spatially limited to a single dimension.

While the previous methods (D${}_{2}$O, IR and spatial selective pulses) utilized modification of the longitudinal relaxation (T${}_{1}$), we hypothesized that modification of the transverse relaxation (T${}_{2}$ or T${}_{2}$* for gradient echo sequences) of water signal can be an easier, cheaper and more feasible solution. Several studies have exploited contrast agents to reduce the WB T${}_{2}$* relaxation time. Manganese chloride (MnCl${}_{2}$) was used by Delannoy et al. [14] for hyperthermia and Chopra et al. [21] for MR guided high intensity focused ultrasound (HIFU). Allen et al. have proposed the use of suspending iron oxide nanoparticles (SPIO) to suppress water bath signal for MRgFUS surgery and reported that SPIO doped water weakly attenuates acoustic waves [22]. Although these studies showed promising results, no data are available on their effect on electromagnetic material properties, which are pivotal for efficient MR guided RF hyperthermia. Also, the effect of additives to the water on therapy and the optimal concentration or compound has never been researched.

In this study, we investigated the benefit of T${}_{2}$* reducing additives in terms of MRI signal reduction while maintaining RF properties for different solutions aimed at their application in the WB during MRI guided RF hyperthermia treatments. Water solutions were prepared with compounds that are known for their T${}_{2}$* reducing properties; CuSO${}_{4}$, MnCl${}_{2}$, and Fe${}_{3}$O${}_{4}$. We measured electromagnetic properties 50–600 MHz range and T${}_{2}$* relaxation rates at 1.5 T. Through multi-physics simulations, we evaluated the predicted effect on the power deposition pattern when the WB filling electromagnetic properties changed to the measured ones for two MR compatible hyperthermia applicators. Further, we identified the compounds and concentrations that fulfill the criteria for power efficiency, power deposition patterns and MR images. Finally, the suitable solutions were experimentally tested in a clinical setup of the prototype MRcollar [23] and their effect on MRT precision were assessed.

## 2. Materials and Methods

### 2.1. Requirements for the Water Bolus Fluid

An ideal WB for MRI guided hyperthermia treatments should have similar electromagnetic properties as deionized water while producing no or limited MRI signal and artifacts. Demineralized water filled WB are generally used because of its low losses at the working frequency of hyperthermia applicators, e.g., at 434 MHz the electrical conductivity is equal to 0.04 S/m. Increase in conductivity results in an increase of the power needed to achieve a therapeutic specific absorption rate (SAR) level within the tumor. At Erasmus MC, available amplifiers can provide maximum of 1800 W of RF power. Hence total required power should be below the maximum available power. However, the ideal WB solution should be energy efficient and should not cause a drastic power requirements. The requirements for MRI invisible WB can be achieved when the signal-to-noise ratio (SNR) level of the WB signal drops to 1. Currently in our clinic, dual echo gradient echo sequence with the body coil utilized for receiver has been used for MRT with echo times 4.8 ms and 19.1 ms [24]. Hence, for our setup, we desire SNR of the WB signal should be 1 at the echo time 4.8 ms. Since this will ensure the SNR at echo times longer than 4.8 ms to be 1 as well.

### 2.2. Preparation of the Samples

Three different compounds were selected [14,21,22] that are used to modify T${}_{2}$; CuSO${}_{4}$ (Stock# 102791, Merck KGaA, Darmstadt, Germany), MnCl${}_{2}$ (Stock# 244589, Merck KGaA, Darmstadt, Germany), and Fe${}_{3}$O${}_{4}$ (Stock# US7568, US-Nano-Research, Houston, TX, USA). Six different concentrations for each compound were selected: [50, 100, 250, 500, 1000, 1250 mM] for CuSO${}_{4}$, [0.5, 1, 2.5, 5, 12.5, 25 mM] for MnCl${}_{2}$, and [0.25, 0.5, 1, 2.5, 5, 10 mM] for Fe${}_{3}$O${}_{4}$. The temperature of the water bolus during treatment range from 20 to 30 ${}^{{}^{\circ}}$C. The EM and MRI properties in this range is relatively stable and changes less than 1% [25,26]. Therefore, all the measurements were made at 21 ${}^{{}^{\circ}}$C, at the room temperature. The solutions were prepared by diluting the compounds with demineralized water. The samples were stored in 200 mL measurement cups (diameter 60 mm, height 85 mm).

### 2.3. MR Relaxometry Measurements

T${}_{2}$* relaxation times of CuSO${}_{4}$, MnCl${}_{2}$, and Fe${}_{3}$O${}_{4}$ solutions for 1.5 T were measured using a 450 w MR scanner (GE Healthcare, Waukesha, WI, USA ) at 21 ${}^{{}^{\circ}}$C. Data acquisition was made with a multi echo gradient echo sequence with the following sequence parameters: TR = 300 ms, TE = [1.3, 2.8, 4.3, 5.8, 7.3, 8.8, 10.3, 11.8, 13.3, 14.8, 16.3 ms], FOV = 360 mm, NEX = 2, Slice Thickness = 10 mm, Flip Angle = 40${}^{{}^{\circ}}$. The T${}_{2}$* relaxation rates were calculated by fitting a mono-exponential signal decay model [27] using the nonlinear curve fitting function lsqcurvefit of Matlab (R2018b, The MathWorks Inc., Natick, MA, USA). For each sample, a region of interest was chosen manually.

### 2.4. Electromagnetic Property Measurements

The electrical conductivity (σ [S/m]) and relative permittivity (ϵ) were measured with open-ended coaxial probe DAK-12 (v2.4; SPEAG, Zurich, Switzerland) with a ZNC3 vector network analyzer (Rhode & Schwarz, Munich, Germany). The system calibration was performed using the open and short, and demineralized water at room temperature as load. The samples were placed in 200 mL measurement caps (diameter 60 mm, height 85 mm) and measured 8 times in the frequency range of 50–600 MHz with 1 MHz steps at 21 ${}^{{}^{\circ}}$C. The VNA was recalibrated before each measurement.

### 2.5. Effects on Power Deposition Pattern

The effect of the change in the electromagnetic properties of the WB were evaluated for two MR compatible hyperthermia applicators: the Sigma Eye applicator of the BSD2000-3D/MR system (PYREXAR Medical, West Valley City, UT, USA) [28] and our in-house developed MR-compatible head and neck hyperthermia applicator (MRcollar) [23]. The Sigma Eye consists of 12 dipole antenna pairs operating at 100 MHz and this device is used for deep loco-regional hyperthermia treatments in pelvis region. The WB of this applicator encloses the abdominal region of the patient in treatment configuration. The MRcollar is a twelve-channel applicator and consists of two moon-shaped halves. Modified Yagi-Uda antennas operating at 434 MHz are employed in this applicator [29]. Hyperthermia treatment planning was performed for these applicators for models of two patients that were treated with pelvis or head and neck hyperthermia, respectively. In our simulations, the electromagnetic properties of the WB were changed to those measured for the different solutions. Electromagnetic field distributions were computed per antenna using Sim4Life (v.5.0.1, Zurich MedTech, Zurich, Switzerland) and normalized to 1 W radiated power. Then, the field was optimized using Matlab-based in-house developed adaptive hyperthermia tool VEDO [30]. The effect on the power deposition patterns were evaluated using hyperthermia treatment planning (HTP) parameters target-to-hotspot quotient (THQ) and the target coverage of the 50% iso-SAR volume (TC50) [31,32]. To calculate the effect on power efficiency, total input power was increased until the maximum predicted temperature in the healthy tissue reached 44 ${}^{{}^{\circ}}$C. This power was then used to compare the effect of the change in measured dielectric property for each solution on power efficiency, i.e., heat loss in the WB.

### 2.6. Effects on MRT Precision

The samples that satisfied the requirements were tested in a representative treatment setup using the MRcollar. The other MR compatible applicator available in Erasmus MC the Sigma Eye applicator is in clinical use and the WB cannot be instantly changed, whereas the MRcollar is an experimental prototype and has exchangeable WB. The WB of the right MRcollar shell was used to test the effect of these samples. For all cases, the WB of the left MRcollar shell was filled with demineralized water and the water was not circulated. The in-house developed ADAM phantom (T${}_{1}$: 820 ms; T${}_{2}$: 37 ms), representing the morphology of an average head and neck patient, was scanned with the clinically used MRT sequence for deep hyperthermia treatments (SNR 85 dB) [24]: dual echo gradient echo sequence, 620/4.8/19.1 ms, flip angle 40${}^{{}^{\circ}}$, slice thickness 10 mm, slice spacing 22 mm, 5 slices, FOV 360 × 360 mm${}^{2}$, matrix size 256 × 256, NEX 1. Images were acquired continuously under three different conditions for a total of 15 min: without water circulation between 0–5 min, during the water circulation between 5–10 min (maximum flow rate 1.5 L/min), and after water circulation was stopped between 10–15 min. Furthermore, a reduced FOV (360 × 270 mm${}^{2}$) scan was also tested to show the potential effects of WB signal aliasing. MRT maps were calculated using the proton resonance frequency shift method and applying background drift correction using four regions of interest at the edges of the phantom. Since no heating pulses were applied during the experiment, the expected measured temperature change both temporally and spatially was 0 ${}^{{}^{\circ}}$C. Using this assumption, MRT precision per voxel was calculated by calculating the standard deviation over all PRFS temperature measurements.

## 3. Results

In Table 1, a summary of all results is presented. In the following sections, each measurement will be investigated in detail.

### 3.1. MR Relaxometry Measurements

**Figure 1:**
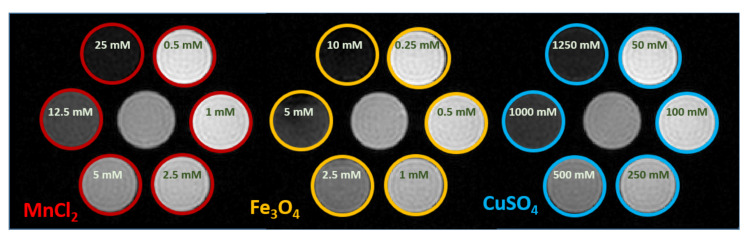
Grayscale MR magnitude images acquired with multi echo gradient echo sequence for different MnCl${}_{2}$, Fe${}_{3}$O${}_{4}$, and CuSO${}_{4}$ concentrations at echo time of 1.3 ms. Complete signal suppression were achieved for each compound for the sample with the highest concentration.

In Figure 1, example magnitude images acquired with multi echo gradient echo sequence at echo time 1.3 ms for different solutions are shown. Qualitative analysis on MRI images show it is possible to reach signal void for every compound. The calculated T${}_{2}$* times at 1.5 T are given in Table 1. The values for 1250 mM CuSO${}_{4}$, 25 mM MnCl${}_{2}$ and 10 mM Fe${}_{3}$O${}_{4}$ solutions were omitted because the fitting failed due to low SNR. In order to reduce the WB signal to the noise level, at least 5 mM Fe${}_{3}$O${}_{4}$, 12.5 mM MnCl${}_{2}$, or 1000 mM CuSO${}_{4}$ was required.

### 3.2. Electromagnetic Property Measurements

**Figure 2:**
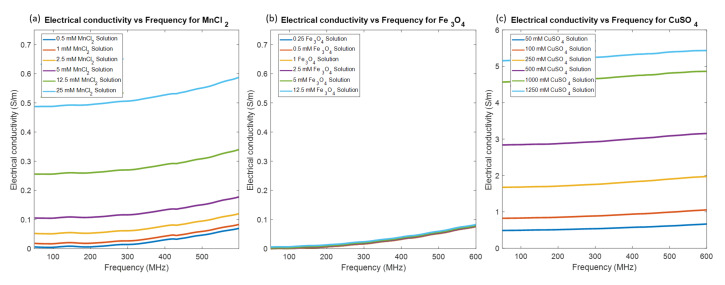
Electrical conductivity over the investigated frequency band for different (**a**) MnCl${}_{2}$, (**b**) Fe${}_{3}$O${}_{4}$, and (**c**) CuSO${}_{4}$ concentrations. Note that the scale in figure (**c**) is different.

In Figure 2, the change in electrical conductivity in the frequency range of 50–600 MHz is illustrated. All three compounds show a linear increase in conductivity with concentration. Addition of Fe${}_{3}$O${}_{4}$ didn’t change the conductivity of demineralized water. However, MnCl${}_{2}$ and CuSO${}_{4}$ had a larger effect. In the case of highest concentration solutions as compared to the demineralized water, conductivity was increased 133 times for CuSO${}_{4}$, and 13 times for MnCl${}_{2}$ at 434 MHz. Permittivity of all solutions were stable and was equal to water relative permittivity in the frequency range (79 ± 0.2 at 434 MHz).

### 3.3. Effects on SAR Patterns and Applicator Efficiency

**Figure 3:**
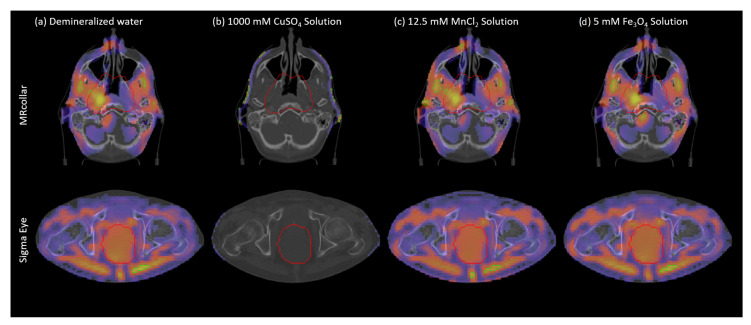
Predicted SAR distributions for models of patients treated with head and neck (MRcollar) and deep pelvis (Sigma Eye) hyperthermia when the WB is filled with (**a**) demineralized water, (**b**) 1000 mM CuSO${}_{4}$ solution, (**c**) 12.5 mM MnCl${}_{2}$ solution, and (**d**) 5 mM Fe${}_{3}$O${}_{4}$.

In Figure 3, example predicted SAR patterns achieved for the solutions satisfying the MRI requirements are shown. In the Figure 3a, the clinical standard demineralized water is visualized. Using that setup (σ: 0.001 S/m at 100 MHz, σ: 0.04 S/m at 434 MHz), THQ of 0.46 and TC50 of 23% for the head and neck patient, and THQ of 0.57 and TC50 of 11% for the deep pelvis patient was achieved. The required power levels to reach maximum efficacy in the treatment were 180 W and 1200 W for MRcollar and Sigma Eye applicators, respectively. As illustrated in the Figure 3 and reported in Table 1, although 1000 mM CuSO${}_{4}$ solution (σ: 4.5 S/m at 100 MHz, σ: 4.8 S/m at 434 MHz) is able to nullify the MRI WB signal, it appears not suitable for hyperthermia purposes due to the high losses. On the other hand, 5 mM Fe${}_{3}$O${}_{4}$ solution (σ: 0.003 S/m at 100 MHz, σ: 0.04 S/m at 434 MHz) had no effect on the SAR pattern and required only a small change in power (ΔW: −37 W for Sigma Eye and ΔW: 11 W for MRcollar) to achieve maximum effectiveness. Finally, it is possible to reach similar predicted SAR patterns with a 12.5 mM MnCl${}_{2}$ solution (σ: 0.001 S/m at 100 MHz, σ: 0.04 S/m at 434 MHz). However, this goes at the cost of additional hot-spots at the skin and total RF-input power needs to be increased by 3000 W for Sigma Eye and 145 W for MRcollar to achieve the same treatment efficacy.

Our investigations reported in the previous three sections show that CuSO${}_{4}$ is not a suitable compound for our aim. On the other hand, both 12.5 mM MnCl${}_{2}$ and 5 mM Fe${}_{3}$O${}_{4}$ solutions appeared suitable for hyperthermia purposes. As such, these have been experimentally tested in the treatment setup.

### 3.4. Effect on MRT Precision

**Figure 4:**
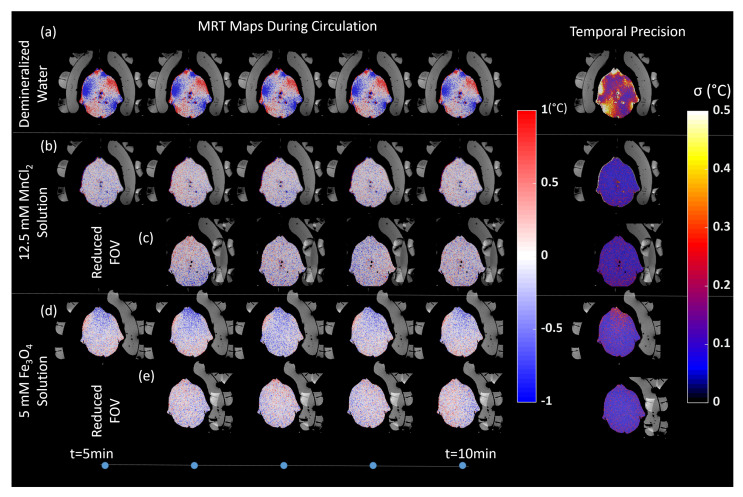
MRT maps and temporal precision map during the water circulation when the applicator right side WB (left in the image) was filled with (first row (**a**)) demineralized water; (second (**b**) & third row (**c**)) 12.5 mM MnCl${}_{2}$ solution full and reduced Field of View, respectively; (fourth (**d**) &fifth (**e**) row) 5 mM Fe${}_{3}$O${}_{4}$ solution full and reduced Field of View, respectively. In the last column, MRT precision per voxel during the water circulation is shown. The expected measured temperature change both temporally and spatially was 0 ${}^{{}^{\circ}}$C. Using this assumption, MRT precision was calculated by calculating the standard deviation over all PRFS temperature measurements. Note that the applicator left side WB (right in the image) was always filled with demineralized water for reference.

In Figure 4, the MRT and the temperature standard deviation (σ) maps acquired during water circulation are shown for demineralized water, 12.5 mM MnCl${}_{2}$ solution, and 5 mM Fe${}_{3}$O${}_{4}$ solution. Note that flow was applied between the 5–10 min. In Table 2, the mean, standard deviation and maximum temperature errors are given for all three cases before, during and after applying (doped) water circulation. The temperature precision on average in the phantom when demineralized water was used in the WB was 0.17 ${}^{{}^{\circ}}$C, 0.70 ${}^{{}^{\circ}}$C, 0.29 ${}^{{}^{\circ}}$C without water circulation, during water circulation, after water circulation respectively. Hence, the flow severely affects MRT precision. Precision improved to 0.15 ${}^{{}^{\circ}}$C, 0.16 ${}^{{}^{\circ}}$C, and 0.26 ${}^{{}^{\circ}}$C when the demineralized water was doped with 12.5 mM MnCl${}_{2}$. The reduction in the FOV made possible by reduction of the signal from the WB further improved precision to 0.11 ${}^{{}^{\circ}}$C when circulation was on. Figure 4c shows the signal aliasing from the antenna modules that are filled with demineralized WB, while there is no aliasing from the right WB (left in the image), which contained MnCl${}_{2}$-doped water. MRT precision was the highest when the 5 mM Fe${}_{3}$O${}_{4}$ solution was used, i.e., 0.09 ${}^{{}^{\circ}}$C, 0.11 ${}^{{}^{\circ}}$C, and 0.11 ${}^{{}^{\circ}}$C (full FOV: without, during and after circulation), and 0.09 ${}^{{}^{\circ}}$C (reduced FOV: during circulation). Figure 4e clearly shows that, while the signal aliasing from the antenna modules are visible, the signal from the WB is absent. A similar improvement in the maximum error values was seen when 5 mM Fe${}_{3}$O${}_{4}$ solution were used instead of demineralized water and MnCl${}_{2}$ solution. The maximum error values were always found for single voxels at the phantom – air interfaces, near the right side of the phantom. This error is mainly caused by the steep phase changes in these interfaces and the partial volume effect. In the clinical scenario, such values are masked by using maximum temperature difference thresholding.

## 4. Discussion

In this study, we determined that doping the water in the WB by (5 mM) Fe${}_{3}$O${}_{4}$ satisfies the needs for MRI signal suppressing while having no effect on the SAR pattern and applicator efficiency. The concentrations of Fe${}_{3}$O${}_{4}$ used in this study do not change the electromagnetic properties of the demineralized water, hence the predicted RF-power deposition patterns and efficiency is the same as in the currently used clinical setup. Local magnetic field inhomogeneity created by Fe${}_{3}$O${}_{4}$ particles causes rapid MR signal decay, therefore it is ideal for MRI signal suppression. In our MRI experiments, this effect resulted in on average 75% improvement in MRT precision. Hence, (1) the substantial improvements in the MRT precision, (2) the absence of an effect on both the predicted SAR and power efficiency and (3) its simple use makes adding Fe${}_{3}$O${}_{4}$ the water in the WB a very elegant solution that can considerably improve MR guided hyperthermia.

Our results show that MnCl${}_{2}$ and CuSO${}_{4}$ can be used for WB signal suppression. However, this comes at the expense of heating efficiency reduction (at least 80% increase in the total input power is required). A WB solution including MnCl${}_{2}$ and reducing the FOV improved the MRT precision by 70% compared to the demineralized water setup. Although this remarkable improvement, energy losses in the WB are high due to the increase in the conductivity. For the head and neck hyperthermia setup, the power demands can be supplied by the power amplifiers available in our clinic. However, the power demands for the deep pelvis hyperthermia applicator is above the total amount that power amplifiers can provide, and hence, unfeasible. In addition, the increased power loss in the WB put additional constraints on the cooling of the water in order to keep the patient comfortable. Additional heat stress caused by the WB is highly unwanted. Lastly, CuSO${}_{4}$ solutions that can nullify MRI signal create a very lossy medium which leads to unacceptable losses in the WB of the RF hyperthermia applicators.

This paper presents the first comprehensive analysis on the T${}_{2}$ shortening agents to be used in the WB for MR RF guided hyperthermia treatments. The results of our work demonstrate the ability of these agents to improve different aspects of the treatment. First, they can reduce temperature measurement errors caused by the water motion. As it has been demonstrated by our results when the demineralized water circulated, the flow severely affects the MRT precision. This effect was largely eliminated with the addition of T${}_{2}$ shortening agents. The reported MRT accuracy of RF hyperthermia devices are in the range of 1 ${}^{{}^{\circ}}$C [13]. However, there is a clear thermal dose effect relation [33,34,35,36] and hotspots in normal tissues still hamper current treatments. Any gain in temperature monitoring will allow to adapt heating settings to improve treatment. These studies also suggest that even small increases in temperature of 0.5 ${}^{{}^{\circ}}$C can lead to improved treatment outcome. Hence, the improvement by the proposed approach will lead to a clinically relevant improvement in treatment monitoring, which will improve treatment reporting and can be used for adapting the treatment. Second, the FOV reduction improves resolution and reduces partial volume effects as well as the scan time, both of crucial importance. This last point, indeed, may result in a shortening of the scan times, increase in data sampling rate, or higher temperature precision by altering the sequence parameters (e.g., by increasing repetition time). At the same time, Fe${}_{3}$O${}_{4}$ solutions have the same electromagnetic properties of the demineralized water hence the utilization of these solutions have no direct tradeoffs for the RF hyperthermia treatments. In addition to these technical improvements, there is another very important benefit due to the possibility to continue water flow: continuity in the cooling of superficial tissues of the patient during heating. This will help to reduce superficial hotspots to improve patient comfort, and consequently will help to reduce patient movements. Moreover, continuous cooling and improved comfort increase the patient’s tolerance to the hyperthermia treatment and therefore provides the clinical condition required to maximize thermal dose to each patient.

In this study, we have shown the potential of the T${}_{2}$ shortening agents using numerical modelling and experimentally in clinically representative setup. However, the actual clinical setup includes components that is not addressed here; mainly the heating RF signal and involuntary patient motion. It is known that for example artefacts from introduction of additional frequencies can occur and image noise increases with increasing power [37]. Although, heating RF signal causes artifacts, its affect will be equal in all cases. Similarly, involuntary motion such as breathing, bowel movement etc. will affect all setups equally. Therefore, while our results can be optimistic for real clinical usage, the potential relative improvement compared to the demineralized water in the WB cannot be underestimated.

The main advantage of the use of demineralized water is its biocompatibility and biodegradability. Fe${}_{3}$O${}_{4}$ nanoparticles are also biocompatible and used for magnetic hyperthermia treatments [38]. An unstudied potential issue is long term degradation of the device due to the additive. If this turns out to be a problem, all the parts of the device in contact with the circulated fluid (antennas, tubing, connectors etc.) might need corrosion inhibition, for instance coating.

In our investigation, we focused on MR guided RF hyperthermia treatments but our findings can also be applied for MR guided HIFU applications. In this case, a WB is used for coupling of acoustic waves and cooling. Removal of WB signal will result in similar improvements in MRT precision as we have shown in this study as shown in [22]. Still, the effect of these compounds on acoustic properties and their effect on losses in the WB should investigated.

## 5. Conclusions

In this work, we have shown that using Fe${}_{3}$O${}_{4}$ nanoparticles doped water instead of the demineralized water in WB can be used to improve MR guided RF hyperthermia treatments. First, MRT precision on average was shown to improve from 0.39 ${}^{{}^{\circ}}$C to 0.09 ${}^{{}^{\circ}}$C using a clinical setup and a patient representative head and neck phantom. Second, the Fe${}_{3}$O${}_{4}$ concentrations required for MR signal suppression of the WB do not alter the electromagnetic properties of the water in the working frequencies of the RF hyperthermia applicators. Besides the possibility of long term impact on the device, there is no tradeoff in terms of heating and imaging when replacing demineralized water by a Fe${}_{3}$O${}_{4}$ solution. Last, doping the water with MnCl${}_{2}$ can also provide similar improvements in MRT precision but it comes with cost of increasing the losses in the WB. In summary, Fe${}_{3}$O${}_{4}$ nanoparticles doped water bolus improves the MRT precision with no performance tradeoffs and has great potential to improve RF hyperthermia treatment monitoring under MR guidance.

## Figures and Tables

**Table 1:** T${}_{2}$* at 1.5 T, conductivity and relative permittivity of demineralized water and different water solutions at 100 and 434 MHz, and their effect on hyperthermia treatment planning parameters target-to-hotspot quotient (THQ), the target coverage of the 50% iso-SAR volume (TC50 [%]) and required power to reach 44 ${}^{{}^{\circ}}$C in the healthy tissue (Power [W]) for two different MR compatible RF hyperthermia devices. T${}_{2}$* values donated with ≈ are not fitted due to low SNR.

		MRcollar					Sigma Eye				
	T2* (ms) **at 1.5 T**	**σ(S/m)** **at 434 MHz**	**ϵ** **at 434 MHz**	**THQ**	**TC50** **(%)**	**Power** **(W)**	**σ (S/m)** **at 100 MHz**	**ϵ** **at 100 MHz**	**THQ**	**TC50** **(%)**	**Power** **(W)**
**Demineralized** **Water**	120	0.04	79.2	0.43	23	180	0.001	79.1	0.57	11	1201
**50 mM** **CuSO${}_{4}$ Solution**	10.8	0.59	82.1	0.39	22	570	0.495	83.5	0.57	2	9564
**100 mM** **CuSO${}_{4}$ Solution**	6	0.95	84.2	0.32	14	1110	0.831	86.0	0.47	2	23948
**250 mM** **CuSO${}_{4}$ Solution**	3.47	1.85	87.5	0.18	0	4761	1.683	89.7	0.16	0	N/A
**500 mM** **CuSO${}_{4}$ Solution**	1.94	3.03	88.1	0.10	0	N/A	2.849	91.1	0.04	0	N/A
**1000 mM** **CuSO${}_{4}$ Solution**	1.09	4.76	83.5	0.05	0	N/A	4.583	88.7	N/A	N/A	N/A
**1250 mM** **CuSO${}_{4}$ Solution**	≈	5.34	79.9	0.04	0	N/A	5.171	85.4	N/A	N/A	N/A
**0.5 mM** **MnCl${}_{2}$ Solution**	11.47	0.03	78.9	0.42	23	177	0.005	78.1	0.57	12	1185
**1 mM** **MnCl${}_{2}$ Solution**	6.27	0.05	78.9	0.43	22	187	0.017	78.2	0.57	10	1337
**2.5 mM** **MnCl${}_{2}$ Solution**	3.77	0.08	79.0	0.43	22	203	0.051	78.2	0.57	5	1656
**5 mM** **MnCl${}_{2}$ Solution**	2.35	0.14	79.0	0.44	22	230	0.105	78.3	0.58	5	2181
**12.5 mM** **MnCl${}_{2}$ Solution**	1.13	0.29	78.9	0.42	21	325	0.256	78.6	0.57	4	4144
**25 mM** **MnCl${}_{2}$ Solution**	≈	0.53	79.0	0.40	22	503	0.488	78.8	0.57	2	9734
**0.25 mM** **Fe${}_{3}$O${}_{4}$ Solution**	6.42	0.04	79.2	0.42	22	181	0.001	78.9	0.56	10	1236
**0.5 mM** **Fe${}_{3}$O${}_{4}$ Solution**	4.1	0.04	79.2	0.42	22	179	0.001	78.9	0.57	7	1105
**1 mM** **Fe${}_{3}$O${}_{4}$ Solution**	2.71	0.04	79.2	0.42	23	181	0.001	78.9	0.57	6	1132
**2.5 mM** **Fe${}_{3}$O${}_{4}$ Solution**	1.44	0.04	79.2	0.43	22	183	0.002	78.9	0.57	9	1159
**5 mM** **Fe${}_{3}$O${}_{4}$ Solution**	1.07	0.04	79.2	0.43	22	191	0.003	78.9	0.56	10	1163
**10 mM** **Fe${}_{3}$O${}_{4}$ Solution**	≈	0.05	79.2	0.43	22	186	0.006	78.9	0.57	7	1158

**Table 2:** Mean, standard deviation and maximum temperature errors before water circulation, during water circulation, after water circulation when demineralized water, 12.5 mM MnCl${}_{2}$ solution, and 5 mM Fe${}_{3}$O${}_{4}$ solution is used in the WB.

		Demineralized Water	12.5 mM MnCl${}_{2}$ Solution		5 mM Fe${}_{3}$O${}_{4}$ Solution	
		Full FOV	Full FOV	Reduced FOV	Full FOV	Reduced FOV
**Before**	Mean error (${}^{{}^{\circ}}$C)	−0.06	−0.06		−0.06	
**Circulation**	Std (${}^{{}^{\circ}}$C)	0.17	0.15		0.09	
	Max error (${}^{{}^{\circ}}$C)	1.28	1.89		−1.36	
**During**	Mean error (${}^{{}^{\circ}}$C)	0.04	−0.03	−0.05	−0.13	−0.03
**Circulation**	Std (${}^{{}^{\circ}}$C)	0.70	0.16	0.11	0.11	0.09
	Max error (${}^{{}^{\circ}}$C)	41.8	−2.00	−2.05	−1.57	1.32
**After**	Mean error (${}^{{}^{\circ}}$C)	0.20	−0.16		−0.05	
**Circulation**	Std (${}^{{}^{\circ}}$C)	0.29	0.26		0.11	
	Max error (${}^{{}^{\circ}}$C)	28.1	5.40		−2.60	

## Abbreviations

The following abbreviations are used in this manuscript:

RF	Radio frequency
MRI	Magnetic resonance imaging
MRT	Magnetic resonance thermometry
WB	Water bolus
IR	Inversion recovery
MRgFUS	Magnetic resonance guided focused ultrasound surgery
HIFU	High intensity focused ultrasound
SPIO	Suspending iron oxide nanoparticles
SAR	Specific absorption rate
SNR	Signal-to-noise ratio
THQ	Target-to-hotspot quotinent
TC50	Target coverage of the 50% iso-SAR volume
